# The dominant lineage of an emerging pathogen harbours contact-dependent inhibition systems

**DOI:** 10.1099/mgen.0.001332

**Published:** 2025-01-24

**Authors:** Cristian V. Crisan, Joanna B. Goldberg

**Affiliations:** 1 Department of Pediatrics, Division of Pulmonary, Asthma, Cystic Fibrosis, and Sleep, Emory University School of Medicine, Atlanta, Georgia, USA; 2 Emory+Children’s Center for Cystic Fibrosis and Airway Disease Research, Emory University School of Medicine, Atlanta, Georgia, USA

**Keywords:** contact-dependent inhibition, emerging pathogen, *Stenotrophomonas maltophilia*

## Abstract

Bacteria from the *Stenotrophomonas maltophilia* complex (Smc) are important multidrug-resistant pathogens that cause a broad range of infections. Smc is genomically diverse and has been classified into 23 lineages. Lineage Sm6 is the most common among sequenced strains, but it is unclear why this lineage has evolved to be dominant. Antagonistic interactions can significantly affect the evolution of bacterial populations. These interactions may be mediated by secreted contact-dependent proteins, which allow inhibitor cells to intoxicate adjacent target bacteria. Contact-dependent inhibition (CDI) requires three proteins: CdiA, CdiB and CdiI. CdiA is a large, filamentous protein exported to the surface of inhibitor cells through the pore-like CdiB. The CdiA C-terminal domain (CdiA-CT) is toxic when delivered into target cells of the same species or genus. CdiI immunity proteins neutralize the toxicity of cognate CdiA-CT toxins. We found that all complete Smc genomes from the Sm6 lineage harbour at least one CDI locus. By contrast, less than a quarter of strains from other lineages have CDI genes. Smc CdiA-CT domains are diverse and have a broad range of predicted functions. Most Sm6 strains harbour non-cognate *cdiI* genes predicted to provide protection against foreign toxins from other strains. Finally, we demonstrated that an Smc CdiA-CT toxin has antibacterial properties and is neutralized by its cognate CdiI.

## Data Summary

All genomes examined here are publicly available on the National Center for Biotechnology Information. Genome accession numbers and details are presented in Table S1. Plasmids and primers are presented in Tables S2 and S3, respectively. All plasmids and bacterial strains used in this study are available upon request. Four supplementary tables and four supplementary figures are available in the online version of this article.

**Impact Statement**

Our genomic analyses provide evidence for a biological mechanism that could have shaped the diversity and evolution of an emerging, multidrug-resistant bacterial pathogen. The presence of contact-dependent inhibition (CDI) genes in isolates across the globe highlights the importance of this apparatus as a weapon in the antibacterial arsenal of *Stenotrophomonas maltophilia* complex (Smc). We propose that Smc strains from the Sm6 dominant lineage that acquired CDI genes gained a competitive advantage that allowed them to eliminate related cells. Proteins like the antibacterial toxin we identified and studied here could aid in the design of novel therapeutics.

## Introduction

*Stenotrophomonas maltophilia* is a ubiquitous Gram-negative bacterium isolated from natural environments like soils, waters and plants and from anthropogenic sources like hospitals [1,2]. The bacterium is increasingly recognized as a serious human pathogen that can cause lung, blood, skin and brain infections [2,5]. Bacteraemia patients can have mortality rates greater than 65% [6]. People with conditions such as cancer, cystic fibrosis (CF), chronic obstructive pulmonary disease (COPD) and coronavirus disease 2019 (COVID-19) are especially vulnerable, but healthy individuals are also at risk [7,12]. *S. maltophilia* infections in people with CF or COPD are associated with a higher risk of death and hospitalization [8,10]. Most isolates are multidrug-resistant and difficult to eradicate [1,13]. *S. maltophilia* is listed among the top ten multidrug-resistant bacterial pathogens that cause infections in intensive care units [14]. This pathogen had the highest rates of antibiotic resistance among bacteria isolated from COVID-19 patients and was the most frequent Gram-negative carbapenem-resistant bacteria associated with bloodstream infections [5,11].

*S. maltophilia* isolates display great genomic diversity and have been classified into 23 lineages [15]. Lineage Sm6 (also denoted as *sensu stricto*) is the most common (approximately one-third of all sequenced genomes) and is associated with human infections [15]. Isolates from the *sensu lato* group (which include lineages Sm1–Sm18 but not Sm6) are more closely related to Sm6, while Sgn1–Sgn4 lineages are the most distantly related [15]. The average nucleotide identity (ANI) between genomes from the same lineage is >95 % [15]. Together, these 23 lineages are referred to as *Stenotrophomonas maltophilia* complex (Smc) [15]. Little is known about the molecular factors that might have contributed to the evolution of the Smc.

Antagonistic bacterial interactions can have profound effects on the evolution of microbial communities and may promote the diversification of populations [16,23]. Bacteria are especially likely to engage in competitive behaviours with other bacteria that have similar metabolic pathways [20]. Antagonistic interactions may be mediated by secreted diffusible factors or by contact-dependent inhibition systems that translocate toxic proteins from inhibitor cells into target cells [17,24,27].

Contact-dependent inhibition (CDI) systems (also referred to as Type Vb secretion systems) are molecular weapons used by Gram-negative bacteria to translocate toxins into adjacent cells [28,33]. In general, inhibitor cells use CDI to deliver toxins into related bacterial competitors from the same species or genus [28,34, 35]. CDI systems require three proteins: CdiA, CdiB and CdiI [28]. CdiA is a large (~2000–5000 amio acid (aas)), filamentous protein with a stick-like structure that harbours repeated filamentous haemagglutinin FHA1 and FHA2 regions and variable toxic CdiA C-terminal domains (CdiA-CT) with diverse biochemical functions [28,36,38]. CdiB is an outer membrane protein that facilitates CdiA export to the inhibitor’s cell surface [28]. Once exported, CdiA binds to a receptor on the target cell membrane to deliver the CdiA-CT toxin [34,39, 40]. The identity of the target cell’s receptor determines the specificity of CdiA toxins [34]. CdiI proteins neutralize their cognate CdiA-CT toxins and prevent killer cells from self-intoxication [36]. CDI proteins are usually encoded in operons that have a conserved *cdiB*, *cdiA* and *cdiI* gene order [28]. Orphan, truncated *cdiA* genes can also be found downstream of CDI operons [41]. These orphan sequences lack secretion signals and may serve as reservoirs for toxins that can be recombined into full-length *cdiA* genes [41].

Using publicly available Smc genomes, we observed that all isolates from the major Sm6 lineage harbour at least one CDI locus. By contrast, less than a quarter of *sensu lato* and none of the Sgn genes have CDI genes. Smc CdiA-CT toxic domains are highly diverse, but full-length CdiA proteins can be classified as Type I, Type II-1 and Type II-2 based on their amino acid (aa) sequences. CdiA proteins from these types are encoded by genes found at distinct but conserved genomic locations. We determined that specific CdiA-CT toxins are restricted based on their putative functions to either Type I, II-1 or II-2 CdiA proteins. Smc strains that harbour *cdiA* genes can also possess multiple non-cognate *cdiI* genes that encode proteins predicted to neutralize CdiA-CT toxins distinct from the ones they possess. Finally, we demonstrate that a CdiA-CT toxin from an Smc isolate has antibacterial properties and is neutralized by its cognate CdiI. Taken together, our results provide evidence that CDI genes are ubiquitous among isolates from the major *sensu stricto* Smc lineage and could have played important roles in the evolution and genomic diversity of this emerging pathogen.

## Methods

### Smc CDI genome analyses

Smc genomes were obtained from the National Center for Biotechnology Information (NCBI) in October 2023. To ensure the accurate discovery of large CdiA proteins with repetitive domains, complete Smc genomes were analysed. Genomes with less than 10× coverage, with more than one contig or chromosome or with frameshifted proteins, were excluded. Smc strain and genome details are listed in Table S1 (available in the online Supplementary Material). An ANI was performed on the genomes using FastANI (v1.33) [42] and ANIclustermap (v1.2.0) (available at https://github.com/moshi4/ANIclustermap). The phylogenetic tree generated by the ANI analysis was visualized in iTol (v5) [43]. For some CDI loci, the *cdiI* gene was not annotated automatically; additional analyses of the sequences downstream of *cdiA* were conducted to identify *cdiI* genes using the NCBI ORFfinder (available at https://www.ncbi.nlm.nih.gov/orffinder).

### Protein annotations, predictions and alignments

Smc genomes were annotated using Prokka (v1.14.5) [44]. blastp searches with default parameters (BLOSSUM62 matrix) were performed against annotated Smc proteins using CdiA protein sequences from *Burkholderia pseudomallei* strain 1026b (UniProt I1WVY3) and *Escherichia coli* strain NC101 (UniProt P0DSI1) [37,45]. Hits with <30% query coverage or with <1000 aas were discarded to avoid detection of other filamentous proteins or truncated CdiA proteins encoded by orphan *cdiA* genes. Genes encoding CdiA proteins were confirmed to be located downstream of genes predicted to encode CdiB proteins. The functions of proteins encoded by genes found upstream of each locus were predicted using InterPro (v101) [46].

The last C-terminal 150 aas of each identified Smc CdiA (CdiA-CT) were extracted. Protein functions were predicted using the NCBI Conserved Domain Search (v3.21), HHpred (v57c8707149031cc9f8edceba362c71a3762bdbf8), Phyre2 (v2.0) and InterPro (v101) [46,49]. CdiA-CT for which none of the algorithms above predicted a clear function were designated as ‘Unknown/Other’. The aa sequences of CdiA-CT, full-length CdiA or full-length CdiB were aligned using clustal Omega (v1.2.4), and the obtained per cent identities were used to create matrices in Heatmapper (v1) [50,51]. A threshold of 50% identity was used to classify proteins in different groups. muscle (v3.8.425) protein alignments were performed for CdiA-CT^Smc1^ and CdiA-CT^E479^ [52].

### Cloning and site-directed mutagenesis

Standard molecular biology protocols were used as recommended by manufacturers. The final 900 nt of the *cdiA^Smc1^* gene sequence (*cdiA-CT^Smc1^*) from Sm6 strain CCV131 were amplified using VeriFi polymerase (PCR Biosystems, catalogue #PB10.42) and cloned into the pET-28a(+) expression vector (Millipore Sigma, catalogue #69864) using Gibson Assembly (New England Biolabs, catalogue #E2611) [53].

Site-directed mutagenesis was conducted by running PCRs with VeriFi polymerase to introduce alanine aa substitutions for the indicated aas in the wild-type *cdiA-CT^Smc1^* sequence from the pET-28a(+) vector. PCR products were digested with DpnI (New England Biolabs, catalogue #R0176), treated with T4 Polynucleotide Kinase (New England Biolabs, catalogue #M0201) and T4 Ligase (New England Biolabs, catalogue #M0202) and transformed into NEB 5-alpha (New England Biolabs, catalogue #C2987). All plasmids were maintained in *E. coli* NEB 5-alpha cells, and sequences were confirmed by Nanopore sequencing (Plasmidsaurus). Plasmids and primers are listed in Tables S3 and S4, respectively.

### *E. coli* growth inhibition experiments

Competent *E. coli* BL21(DE3)pLysS cells (which express the T7 lysozyme) (Millipore Sigma, catalogue # 69451) were transformed before each experiment with pET-28a(+) or the indicated pET-28a(+)-derived vectors. Cells with plasmids were selected after overnight growth at 37 °C on LB plates containing chloramphenicol (50 µg ml^−1^), kanamycin (30 µg ml^−1^) and 0.2% glucose. Single colonies were inoculated in LB media containing chloramphenicol (50 µg ml^−1^), kanamycin (30 µg ml^−1^) and 0.2% glucose. Cultures were incubated at 37 °C with shaking overnight, washed with fresh LB medium and diluted into media containing chloramphenicol (50 µg ml^−1^), kanamycin (30 µg ml^−1^) and either 0.2% glucose or 50 µM IPTG. Cells were grown with shaking at 37 °C in 96-well plates, and OD_600_ readings were measured using a BioTek Synergy H1 spectrophotometer. Data represent averages from at least three biological replicates.

## Results

### All isolates from the Sm6 lineage encode full-length CdiA proteins

**Fig. 1.**
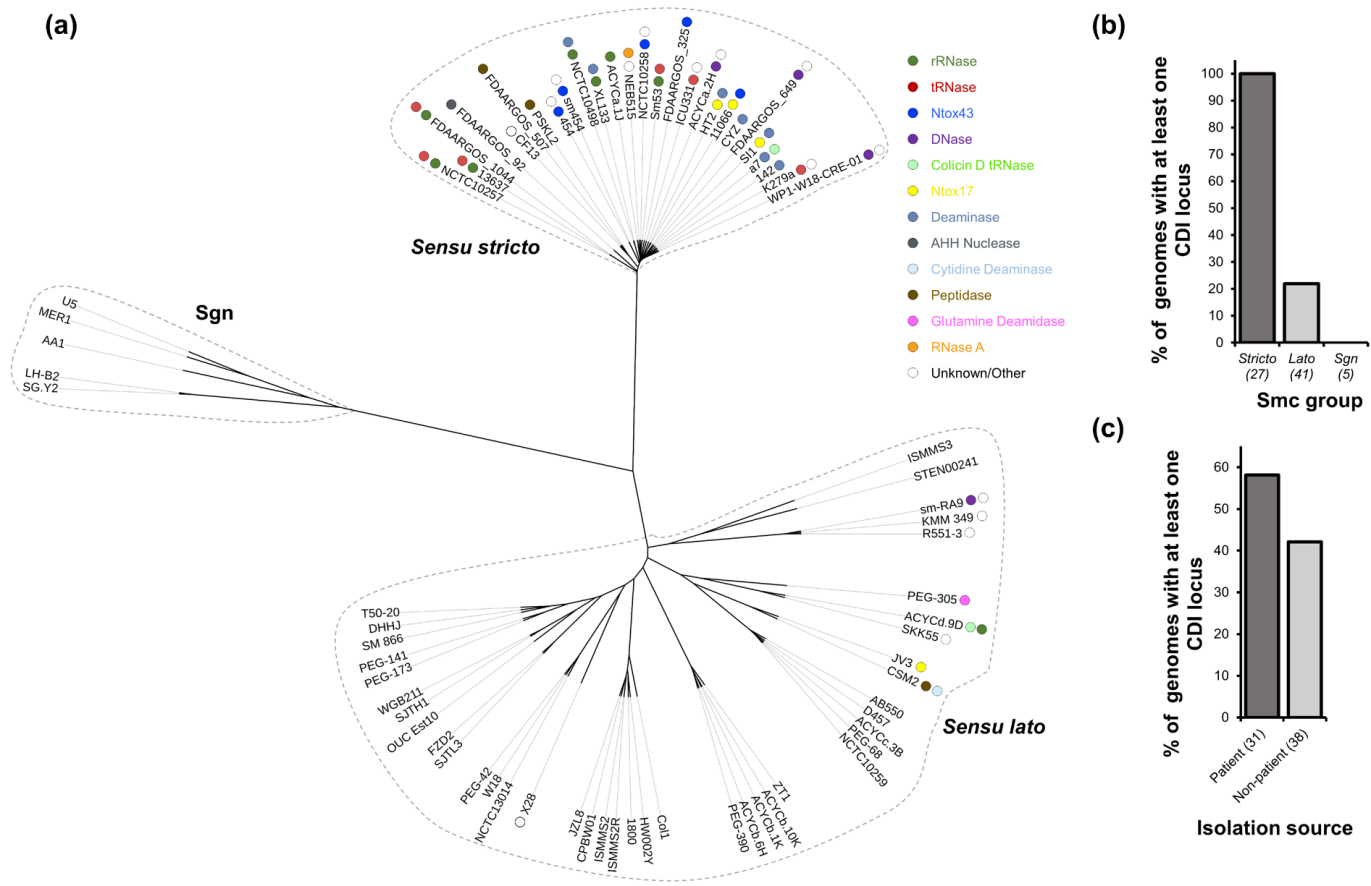
CDI genes are predominantly found in the major Smc lineage. (**a**) 73 Smc complete genomes were retrieved from NCBI, annotated with Prokka, and an ANI analysis was performed using FastANI [42,44]. A phylogenetic tree based on the ANI was visualized in iTol [43]. A blastp search with default parameters was used to identify CdiA proteins. Strain names are depicted at the branch tips. Each circle near strain names represents a different CdiA protein, and colours depict the indicated functions. Protein functions were predicted using the NCBI Conserved Domain, InterPro, Phyre2 and HHPred [46,49]. (**b**) Percentage of Smc genomes from the *sensu stricto*, *sensu lato* and Sgn groups that harbour at least one CDI gene locus. (**c**) Percentage of Smc strains from patient or non-patient sources that encode at least one CDI gene locus. Four strains with unknown isolation sources were excluded from this analysis.

We retrieved 73 complete Smc genomes available from the NCBI and performed an ANI analysis based on their full-length genome sequences (Fig. 1a, Table S1). As expected, the Sm6 lineage was the most common with ~37% of the analysed genomes classified as *sensu stricto* (Fig. 1a) [15]. Other genomes belonged to either *sensu lato* (~56 %) or Sgn (~7 %) lineages (Fig. 1a) [15]. We annotated and extracted predicted proteins from these genomes and performed protein homology searches using aa sequences of CdiA proteins from *E. coli* and *B. pseudomallei* [37,45]. We found that all examined *sensu stricto* Smc genomes encode at least one full-length CdiA protein (Fig. 1a, b). By contrast, only ~22% of the analysed *sensu lato* and none of the Sgn genomes harbour genes encoding full-length CdiA proteins (Fig. 1a, b). The C-terminal regions of Smc CdiA toxins have diverse predicted functions like tRNases, rRNases, DNases, deaminases and peptidases (Fig. 1a) [37,54,58]. Approximately 58% of genomes analysed here that were obtained from patient sources encode CdiA proteins, while 42% of genomes from non-patient sources encode CdiA proteins (Fig. 1c, Table S1). These results suggest that CDI genes are enriched in *sensu stricto* Smc genomes and are found in strains isolated from clinical and environmental sources.

### Smc CdiA-CT aa sequences cluster into divergent groups

**Fig. 2.**
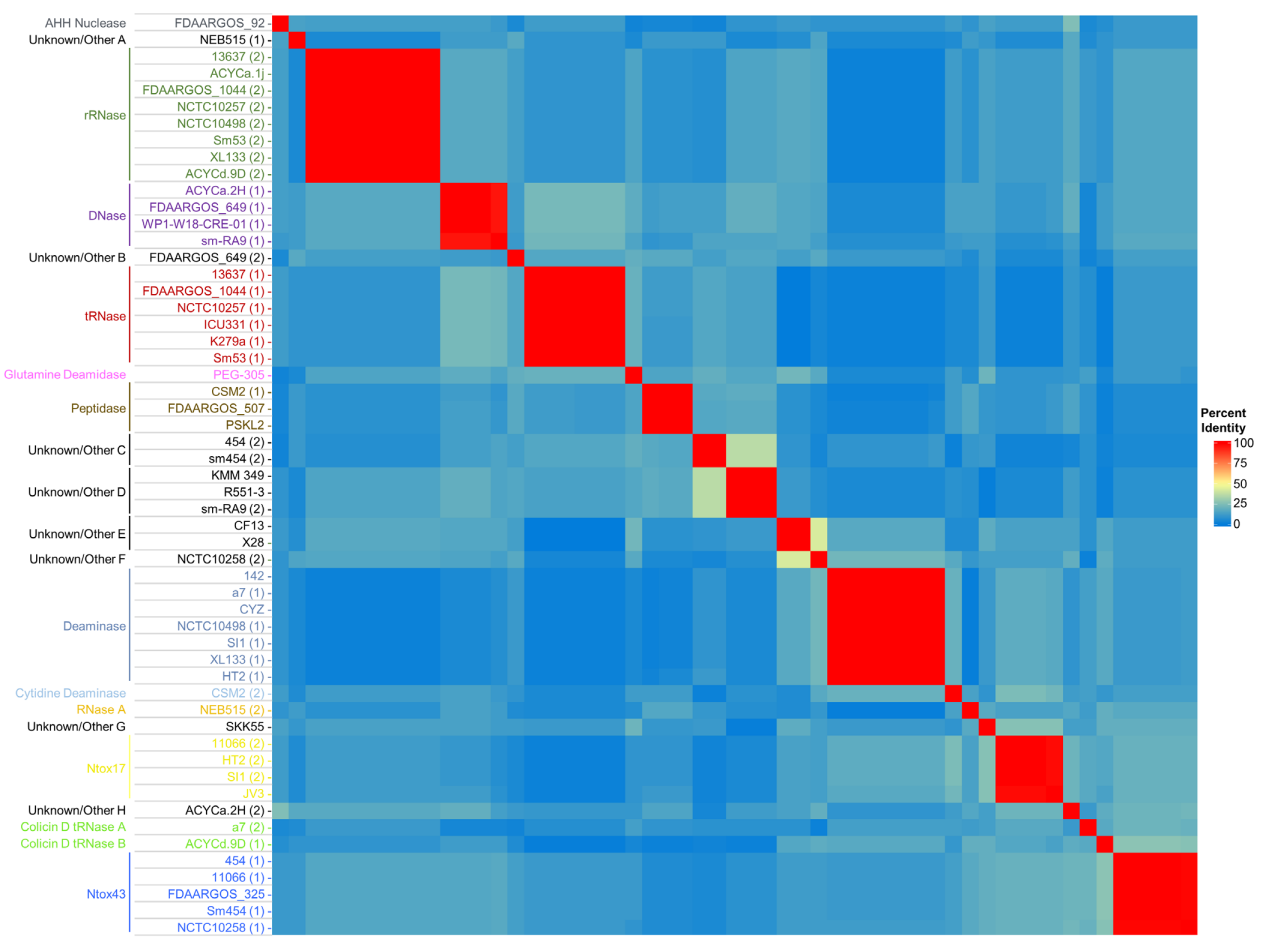
Smc CdiA-CT aa sequences cluster into distinct groups that share limited homology. The final 150 aas of Smc CdiA proteins were obtained and aligned using clustal Omega [50,72]. A matrix was generated from the obtained per cent identities in Heatmapper [51]. Strain names are depicted to the left. For strains that encode two CdiA proteins, numbers in parentheses next to strain names indicate the respective CdiA-CT number.

We analysed the C-terminal regions (the last 150 aas, denoted as CdiA-CT) of the Smc full-length CdiA proteins we identified. CdiA sequences from three strains (ICU331, K279a and WP1-W18-CRE-01) are truncated, and their C-terminal regions were excluded from subsequent analyses (please see the next section, Table S4 and Fig. S1). Smc CdiA-CT sequences that have the same predicted function generally cluster into highly conserved groups based on their aa sequence identity (Fig. 2). By contrast, CdiA-CT sequences from different groups share little homology between them. CdiA-CT designated with Unknown/Other E or F share ~43% homology, indicating that these proteins could have a similar function (Fig. 2). Interestingly, the CdiA-CT aa sequences from Smc strains a7 (2) and ACYCd.9D (1) share limited aa identity (~20 %), but both proteins are predicted to have a function similar to a colicin D tRNase (Fig. 2) [59].

### Smc full-length CdiA and CdiB proteins cluster into distinct types that determine which CdiA-CT toxins can be accommodated

**Fig. 3.**
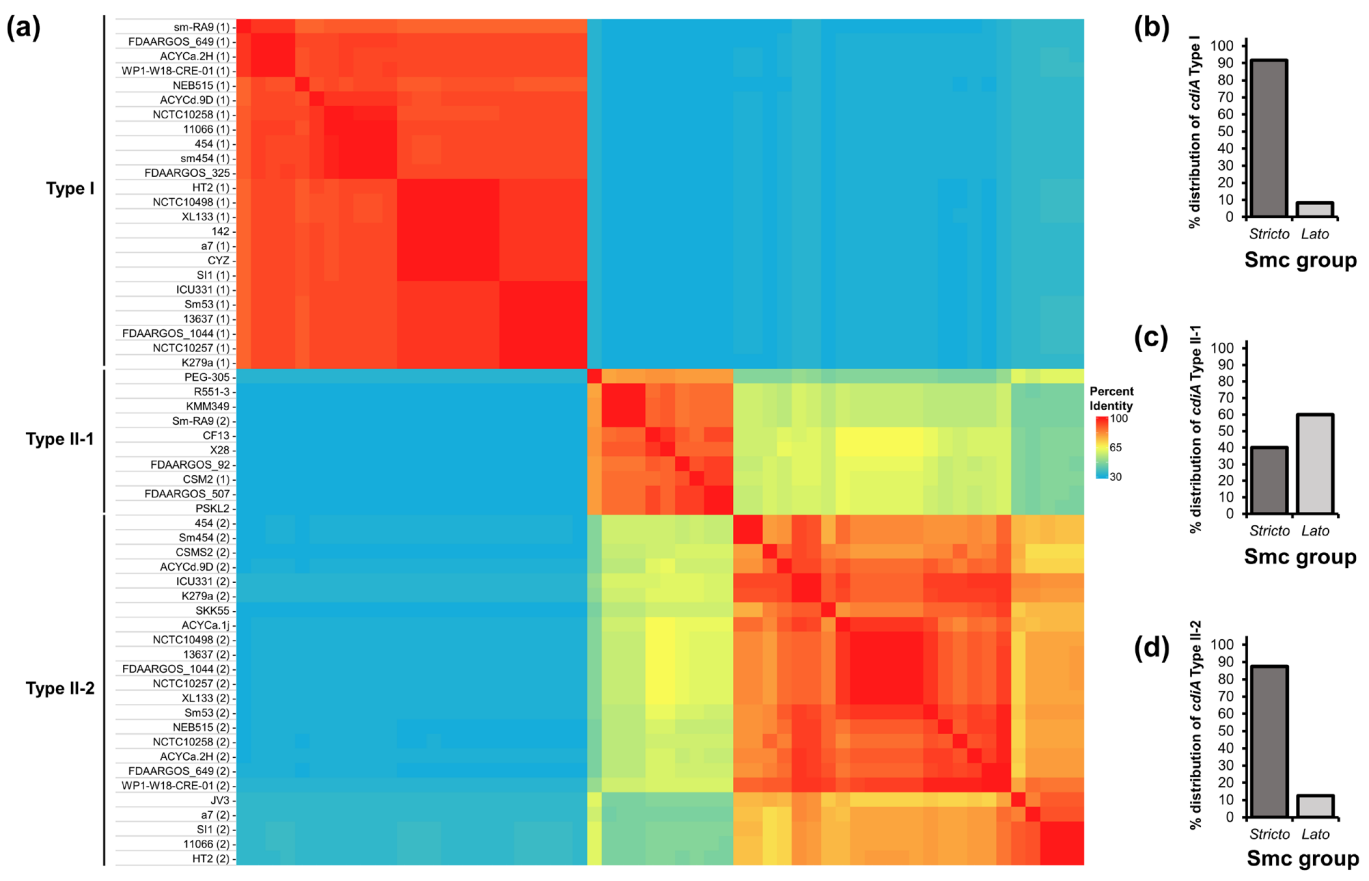
Full-length Smc CdiA proteins can be grouped into Type I, Type II-1 or Type II-2 based on their aa sequences. (**a**) The aas of the full-length Smc CdiA proteins were obtained and aligned using clustal Omega [50,72]. A matrix was generated from the obtained per cent identities in Heatmapper [51]. Strain names are depicted to the left. For strains that encode two CdiA proteins, numbers in parentheses next to strain names indicate the respective CdiA number. (**b**), **(c**), **(d**) The per cent abundance of *cdiA* genes from Type I (**b**), Type II-1 (**c**) and Type II-2 (**d**) was calculated for *sensu stricto* and *sensu lato* Smc genomes.

We performed a homology analysis on full-length Smc CdiA proteins and discovered that aa sequences of CdiA proteins cluster into distinct types: Type I, Type II-1 and Type II-2 (Fig. 3a). The same Type I, Type II-1 and Type II-2 clustering pattern was also observed for CdiB proteins that correspond to their respective CdiA proteins (Fig. S2). CdiA Type I proteins have sizes between 3414 and 3512 aas, CdiA Type II-1 proteins have sizes between 4574 and 5174 aas and CdiA Type II-2 proteins have sizes between 3537 and 4269 aas (Table S2). CdiA Type II-2 proteins from strains ICU331, K279a and WP1-W18-CRE-01 have smaller sizes compared with other proteins from the same type because their C-terminal sequences are truncated (Table S2, Fig. S1). Among the Smc strains we examined, CdiA proteins from both Type I and Type II-2 are found almost exclusively in *sensu stricto* isolates; by contrast, CdiA proteins from Type II-1 are more common in *sensu lato* isolates (Fig. 3b–d).

**Fig. 4.**
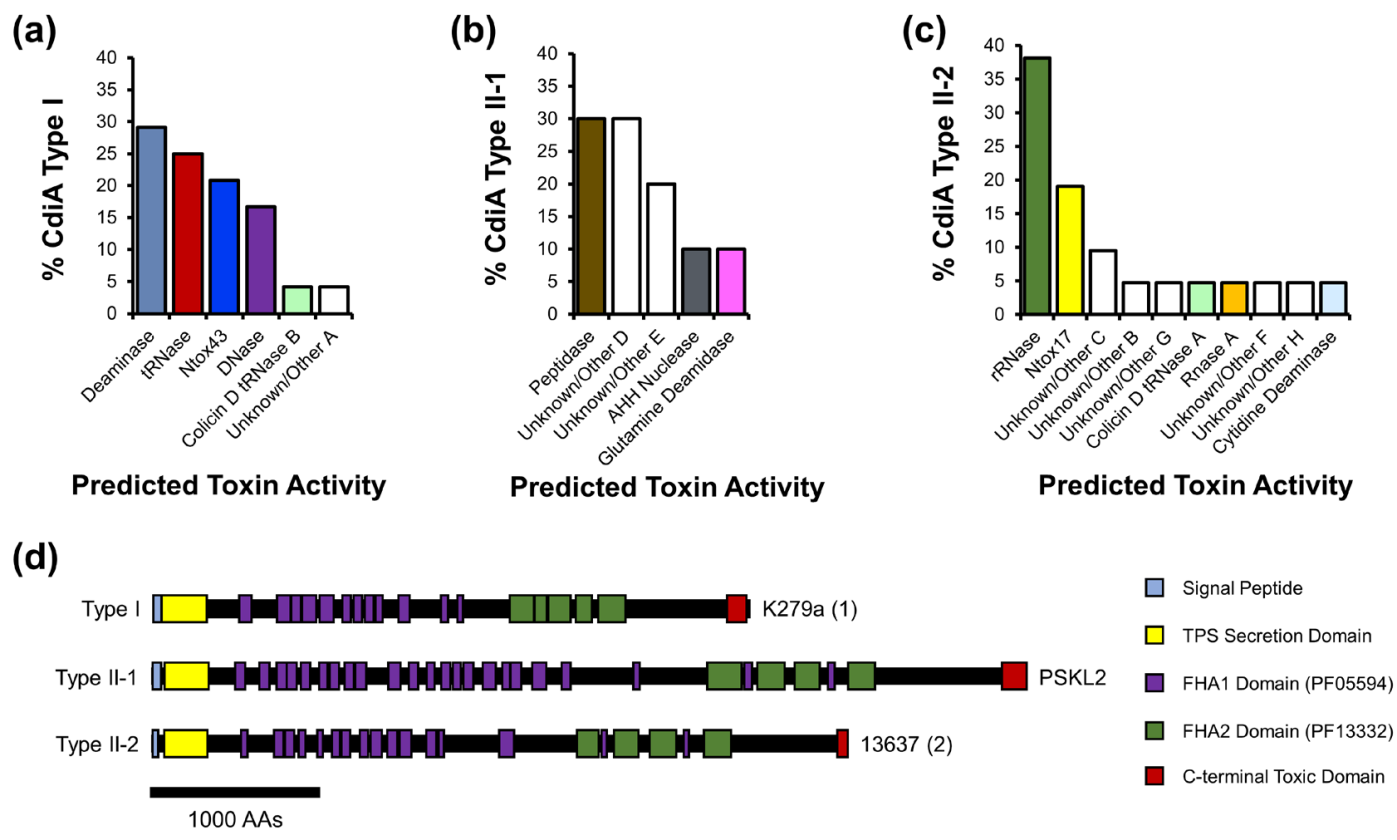
Type I, Type II-1 and Type II-2 Smc CdiA proteins harbour distinct CdiA-CT toxins. (**a**), **(b**), **(c**) The per cent abundance of each CdiA-CT predicted function was calculated for CdiA proteins from Type I (**a**), Type II-1 (**b**) and Type II-2 (**c**). **(d**) Domain architectures of representative CdiA proteins from Type I, Type II-1 and Type II-2 were predicted with InterPro. Strain names are depicted at the right of each protein representation. The scale bar represents 1000 aas.

We next compared the distribution of predicted full-length CdiA-CT toxins among the different CdiA types. We discovered that CdiA-CT toxins are restricted to specific CdiA types and vary in their abundance (Fig. 4a–c). For example, toxins from CdiA Type I proteins include those with putative deaminase, tRNase, Ntox43, DNase, colicin D tRNase B or Unknown/Other A functions (Fig. 4a) [54,57, 59]. Full-length Smc CdiA proteins from different types have similar domain organizations with N-terminal signal peptides (required for export to the periplasm), two-partner secretion domains (required for extracellular export through CdiB), filamentous haemagglutinin FHA1 (PF05594) and FHA2 (PF13332) repeated domains and C-terminal predicted toxins [28].

### Genes encoding CDI proteins from Type I, Type II-1 and Type II-2 are found at distinct but conserved genomic locations

**Fig. 5.**
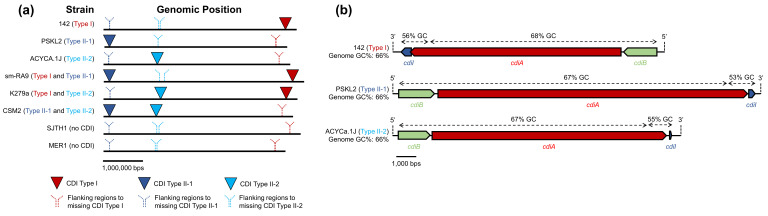
The location of CDI genes is conserved among examined Smc genomes. (**a**) Genomic sequences of CDI genes or genomic regions flanking CDI genes from strains 142 (with CDI Type I), PSKL2 (with CDI Type II-1) and ACYCa.1J (with CDI Type II-2) were aligned to the indicated Smc genomes. (**b**) The genomic organization and orientation of CDI Type I, Type II-1 and Type II-2 operons are depicted. The GC% is displayed above arrows for the indicated regions of the CDI loci.

Since we observed that some Smc strains possess two CDI loci while others only have a single CDI, we sought to determine the genomic location of these loci. We used the representative genomes of Smc strains 142 (with a CDI Type I), PSKL2 (with a CDI Type II-1), ACYCa.1J (with a CDI Type II-2), sm-RA9 (with both CDI Type I and CDI Type II-1), K279a (with both CDI Type I and CDI Type II-2), CSM2 (with both CDI Type II-1 and CDI Type II-2), SJTH1 (from the *sensu lato* group and without CDI) and MER1 (from the Sgn group and without CDI) (Fig. 5a). Strains with Type I, Type II-1 and Type II-2 CDI operons harbour these genes at approximately the same respective genomic location based on their type (Fig. 5a). For genomes that lack CDI operons of specific types, homologous regions flanking CDI genes from strains that possess them are found at similar genomic locations (Fig. 5a). CDI Type I genes are found on the opposite DNA strand compared with Type II-1 and Type II-2 genes (Fig. 5b). The GC% of the last 1000 bp of the *cdiA* gene and *cdiI* gene modules was lower for all three types (~53–56%) compared with the rest of the operons and the overall genomes (~66–68%) (Fig. 5b).

### Sm6 strains can harbour multiple non-cognate *cdiI* genes

**Fig. 6.**
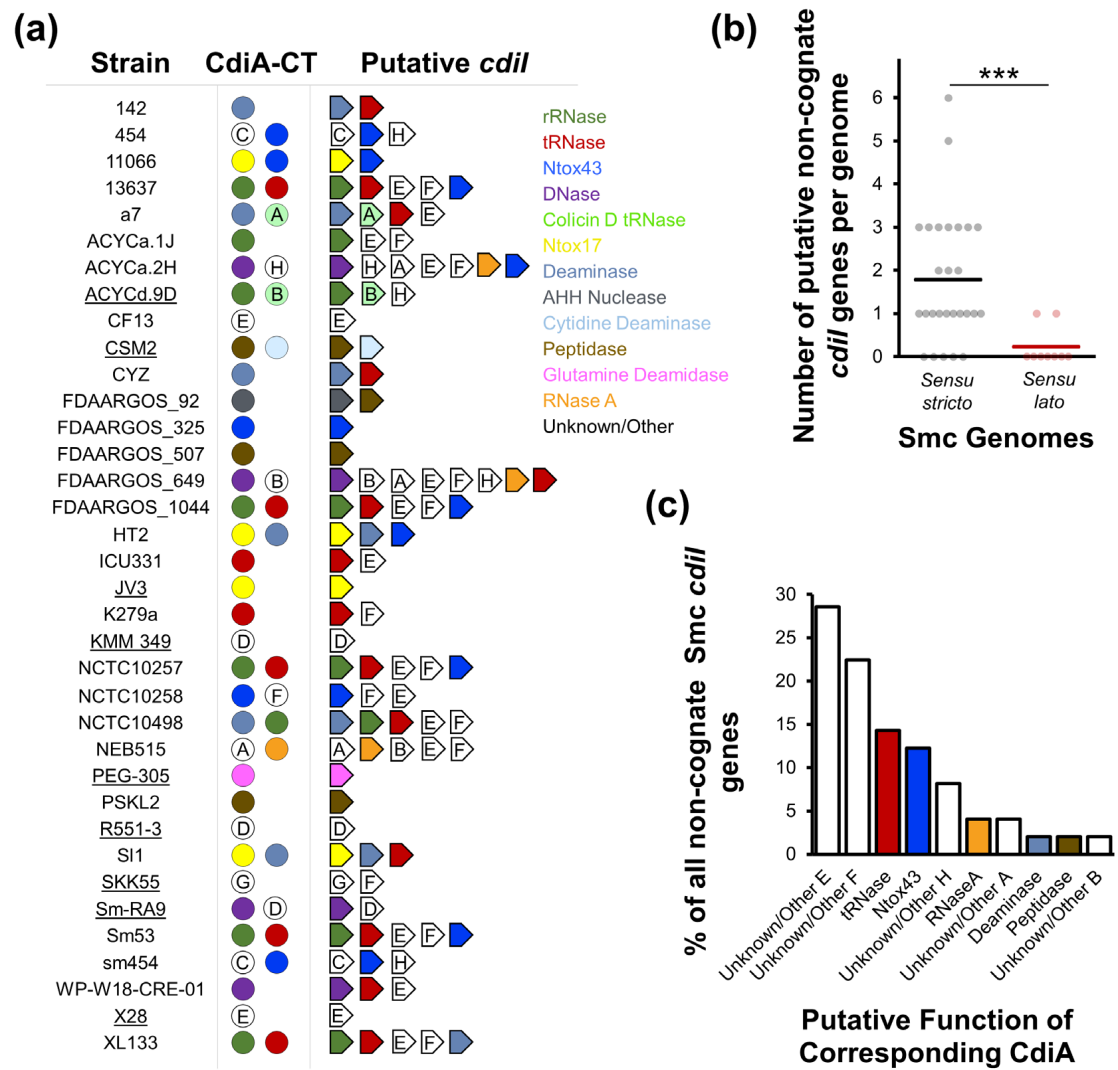
Non-cognate *cdiI* genes are common among Sm6 genomes. (**a**) Putative *cdiI* genes for each *cdiA* from the Smc genomes studied here were annotated, and a blastn search with default parameters was performed using each *cdiI* gene as the query. *cdiI* gene representations are not drawn to scale. *Sensu lato* strains are underlined. Letters inside white arrows depict the group of the corresponding *cdiA* gene. (**b**) The number of non-cognate *cdiI* genes was determined for each Smc *sensu stricto* and *sensu lato* genome analysed in this study. Circles represent the number of *cdiI* genes in each genome. Horizontal black and red bars represent the average numbers of non-cognate *cdiI* genes per genome for *sensu stricto* and *sensu lato* strains, respectively. A Welch’s unpaired t-test was performed to determine statistical significance. ***P<0.001. (**c**) The per cent prevalence of each non-cognate *cdiI* gene was determined for the Smc genomes examined here.

Immunity CdiI proteins are exclusive to and neutralize the toxicity of cognate CdiA toxins specifically [36,37]. Most Smc strains that possess at least one full-length *cdiA* gene harbour non-cognate *cdiI* genes predicted to encode proteins that neutralize the toxicity of different (foreign) CdiA-CT toxins from other strains (Fig. 6a). By contrast, Smc strains that lack full-length *cdiA* genes do not harbour any *cdiI* genes. Sm6 *sensu stricto* strains have significantly more non-cognate *cdiI* genes per genome compared with Smc *sensu lato* strains (Fig. 6b). Putative *cdiI* genes for CdiA proteins with functions annotated as Unknown/Other E or F, tRNase and Ntox43 are the most common non-cognate *cdiI* genes in the Smc genomes we analysed (Fig. 6c). CdiI proteins corresponding to CdiA proteins with functions annotated as Unknown/Other E and F share ~39% aa homology between them and contain a DUF596, which has been previously identified in immunity proteins from polymorphic toxins [57].

### An Smc CdiA-CT displays antibacterial properties with its toxicity neutralized by the cognate immunity protein

**Fig. 7.**
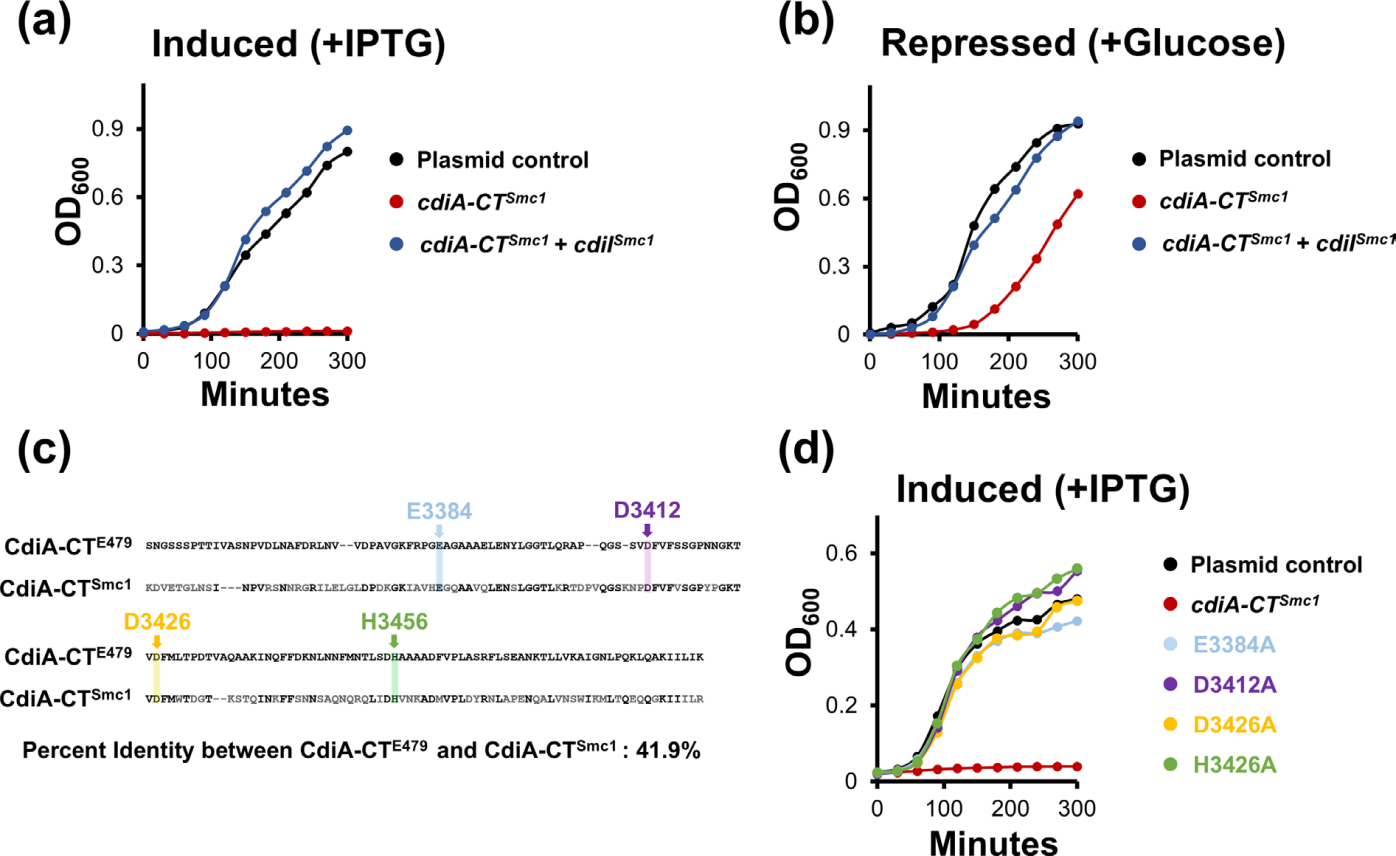
Wild-type CdiA-CT^Smc1^ is toxic to *E. coli* cells but is neutralized by its cognate CdiI^Smc1^ immunity protein. (a) The OD_600_ was measured over time for *E. coli* BL21(DE3)pLysS cells carrying a control plasmid (black line), a plasmid expressing CdiA-CT^Smc1^ (red line) or a plasmid expressing both CdiA-CT^Smc1^ + CdiI^Smc1^ (dark blue line) grown in liquid LB media supplemented with chloramphenicol, kanamycin and 50 µM IPTG. (b) Same as in (a), but cells were grown with 0.2% glucose instead of IPTG. (c) aa muscle alignment of the last 150 aa of CdiA-CT^E479^ and CdiA-CT^Smc1^ [52]. Arrows depict aas from CdiA-CT^Smc1^ that are conserved with the active site aas from CdiA-CT^E479^. (d) The OD_600_ was measured over time for *E. coli* BL21(DE3)pLysS cells carrying a control plasmid (black line), a plasmid expressing CdiA-CT^Smc1^ (red line) or a plasmid expressing CdiA-CT^Smc1^ with an alanine substitution in a predicted active site aa (E3384A, light blue; D3412A, purple; D3426A, orange; H3456A, green). *E. coli* cells were grown in liquid LB media supplemented with chloramphenicol, kanamycin and 50 µM IPTG. Data represent averages from at least three biological replicates.

Since CdiA loci are broadly distributed among Smc *sensu stricto* strains, we sought to provide experimental evidence that a CdiA-CT can inhibit bacterial growth. For this analysis, we focused on an Smc CdiA-CT (denoted as CdiA-CT^Smc1^) predicted with high confidence to adopt a tertiary structure similar to CdiA-CT^E479^, which is a tRNase toxin from *B. pseudomallei* [37,54] (Fig. S3). Putative tRNases are among the most prevalent CdiA-CT toxins among Smc isolates (Figs1a  2) [15]. We cloned the final 900 nt of the *cdiA^Smc1^* gene sequence (*cdiA-CT^Smc1^*) under the control of the T7 promoter from the pET-28a(+) vector and introduced the construct in *E. coli* BL21(DE3)pLysS. Under induced (+IPTG) conditions, *E. coli* BL21(DE3)pLysS cells expressing CdiA-CT^Smc1^ from a plasmid were strongly inhibited compared with cells harbouring a plasmid control (Fig. 7a). By contrast, *E. coli* expressing both CdiA-CT^Smc1^ and the cognate CdiI^Smc1^ immunity protein had growth rates similar to cells expressing plasmid control (Fig. 7a). The CdiI^Smc1^ and CdiI^E479^ immunity proteins are also predicted with high confidence to adopt a similar fold (Fig. S4). *E. coli* with CdiA-CT^Smc1^ alone had delayed growth under repressed (+glucose) conditions, probably due to leaky promoter expression (Fig. 7b).

Although the identity between the sequences of the last 150 aas of CdiA-CT^Smc1^ and CdiA-CT^E479^ is only ~42%, active site aas from CdiA-CT^E479^ are conserved in CdiA-CT^Smc1^ (Fig. 7c) [54]. We introduced alanine substitutions in the predicted active site aas (E3384, D3412, D3426 and H3456) of CdiA-CT^Smc1^ (Fig. 7c). *E. coli* expressing CdiA-CT^Smc1^ with either E3384A, D3412A, D3426A or H3456A mutations had growth rates comparable to *E. coli* cells carrying plasmid controls, even when grown under induced conditions (Fig. 7d). These results indicate that CdiA-CT^Smc1^ is toxic to bacterial cells using a mechanism similar to CdiA-CT^E479^ [54].

## Discussion

There is strong evidence that antagonism plays important roles in the structural organization and evolution of microbial ecosystems [16,19, 20, 22]. Intraspecies competition has been proposed to foster diversity within bacterial populations [22]. Bacteria use an extensive arsenal of weapons to intoxicate competitors, especially those with similar metabolic requirements [16,20]. These antibacterial weapons include diffusible factors (like antibiotics and bacteriocins) and contact-dependent protein secretion systems that deliver toxins into adjacent cells [17,24, 25]. However, little is known about the role of antagonism in shaping the genomic diversity and evolution of Smc.

Previous studies have shown that Smc isolates can utilize antibacterial Type IV secretion systems (T4SS) and Type VI secretion systems (T6SS) to eliminate heterologous bacteria [60,64]. Unlike the T4SS and T6SS, which enable killer cells to eliminate heterologous target bacteria from other genera, CDI systems are generally deployed to inhibit related bacteria from the same species or genus [34,35]. All Smc complete genomes from the Sm6 lineage analysed in this study harbour CDI systems, but few genomes from other Smc lineages carry genes that encode these molecular weapons.

The genomic diversity across Smc isolates is extensive. Gröschel *et al*. found evidence that horizontal gene transfer is frequent among Smc isolates and have speculated that multiple adaptation events could have shaped the evolution of this bacterium [15]. Lineage Sm6 is the most common among all sequenced isolates [15]. We propose that the acquisition of CDI genes could have been an important event in the evolutionary history of Smc and the establishment of Sm6 as the dominant lineage. Strains from the Sm6 lineage that acquired CDI genes likely gained a competitive advantage that allowed them to eliminate closely related Smc strains and establish dominance in their environments. Since CDI systems are found in both patient and non-patient Smc isolates, these genes might be important for competition during both colonization of external environments and during infections (Fig. 1c).

The high variability of Smc CdiA-CT sequences also indicates that strains with CDI loci are under evolutionary pressure to diversify their toxin repertoire (Figs1  2). The majority of Smc CdiA-CT toxins have predicted nuclease functions, but we also identified Cdi-CT toxins for which we were unable to assign a predicted function [54,56,59, 65]. The CdiA-CT domain from Smc strain SKK55 (denoted here as Unknown/Other G) shares limited identity with known CdiA-CT tRNases [65]. Functions assigned to Smc CdiA-CT domains are putative and require further experimental confirmation. The low GC% of *cdiA-CT* and *cdiI* toxin-immunity gene modules (~53–56%) compared with the rest of the genomes (~66–68%) suggests that these regions were acquired relatively recently (Fig. 5b).

Our finding that only some CdiA-CT are found within each CDI type suggests that the structure of CdiA proteins from different types has evolved to accommodate specific CdiA-CT toxins at their C-terminus. Acquiring multiple CdiA types could provide important competitive benefits. It is unclear why CdiA Type II-2 proteins from strains ICU331, K279a and WP1-W18-CRE-01 appear to have lost their C-terminal regions. CDI systems have been previously demonstrated to mediate functions other than interbacterial antagonism, such as adhesion, cell aggregation, virulence and signalling [66,70]. CdiA Type II-2 proteins from strains ICU331, K279a and WP1-W18-CRE-01 could also be used for some of these other functions or their toxic function might not be needed in their respective environments.

The presence of two distinct CDI gene clusters found at different genomic positions in the Smc strains studied here indicates that these systems were probably acquired by independent DNA transfer events. Interestingly, even though our results using genomes from Fig. 5a show that CDI loci of Types I, II-1 and II-2 are found at distinct genomic locations, we did not find any Smc strains that harbour all three CDI types. This is in contrast to some *Burkholderia* strains with four distinct CDI loci but might be explained by a high energy cost associated with maintaining and expressing more than two systems [71].

The presence of non-cognate *cdiI* genes in many Smc genomes provides further evidence that CDI systems are important in conferring competitive advantages. Non-cognate *cdiI* genes could provide protection against attacks from strains that harbour other toxins. On average, isolates from the dominant Sm6 lineage have significantly more non-cognate *cdiI* genes per genome than Smc strains from other lineages (Fig. 6b). In addition to competing against other lineages using CDI toxins, it is possible that Sm6 strains engage in antagonistic interactions among themselves. The high diversity of predicted toxins suggests that *cdiA* genes are under evolutionary pressure to diversify the toxin repertoire they encode. Maintaining an array of non-cognate immunity proteins could be an important survival strategy to defend against non-cognate CDI toxins. The higher incidence of some *cdiI* genes compared with others indicates that some toxins are more effective at eliminating Smc competitors and perhaps maintaining immunity against these toxins could be a survival priority.

We also provide experimental evidence that CdiA-CT^Smc1^ has antibacterial activity, but its toxicity is neutralized by its cognate CdiI protein [54]. CdiA-CT^Smc1^ is predicted with high confidence to adopt a similar structure to CdiA-CT^E479^, even though the two proteins share limited homology (Fig. 7c, Fig. S3) [54]. Furthermore, alanine substitutions in aas that are conserved with aas from the active site of CdiA-CT^E479^ suppress the toxicity of CdiA-CT^Smc1^ [54]. These observations suggest that evolutionary pressures conserved the active sites shared between CdiA-CT^Smc1^ and CdiA-CT^E479^ even though the rest of the two protein domains have changed considerably. K279a, the indicator Sm6 strain, also encodes a CdiA-CT^Smc1^ homolog with putative tRNase function (Fig. 1a).

Taken together, our results demonstrate that CDI systems are broadly distributed across the dominant lineage of Smc and could have played important roles in shaping the evolution of this bacterial pathogen. We observed a great diversity of CdiA-CT toxin sequences and found that Smc strains with at least one CDI locus harbour non-cognate immunity proteins that may help protect against foreign toxins. Additional studies are required to uncover the biochemical functions of uncharacterized CdiA-CT toxins, the mechanism(s) by which CDI genes are acquired by Smc isolates, the regulation of CDI genes in patient and environmental isolates and the potential importance of these systems in mediating virulence. Analysing a larger set of complete Smc genomes as they become available in the future will further expand knowledge about the distribution and diversity of CDI genes in this bacterium. Understanding Smc CDI systems could lead to important insights into the evolution and ecology of Smc and could help the development of new therapies against this multidrug-resistant pathogen.

## supplementary material

10.1099/mgen.0.001332 Uncited Fig. S1.

10.1099/mgen.0.001332 Uncited Table S1.

## Abbreviations

aa: amino acid
aas: amino acids
ANI: average nucleotide identity
CDI: contact-dependent inhibition
CdiA-CT: CdiA C-terminal domain
CF: cystic fibrosis
COPD: chronic obstructive pulmonary disease
COVID-19: coronavirus disease 2019
NCBI: National Center for Biotechnology Information
nt: nucleotide
Smc: *Stenotrophomonas maltophilia* complex
T4SS: type IV secretion systems
T6SS: type VI secretion systems
