# Supplementary material for: The dominant lineage of an emerging pathogen harbours contact-dependent inhibition systems
Source: Microb Genom. 2025 Jan 24;11(1):001332. doi: 10.1099/mgen.0.001332 (PMC11893273; doi:10.1099/mgen.0.001332)
Supplement: Uncited Fig. S1. [file mgen-11-01332-s001.pdf]

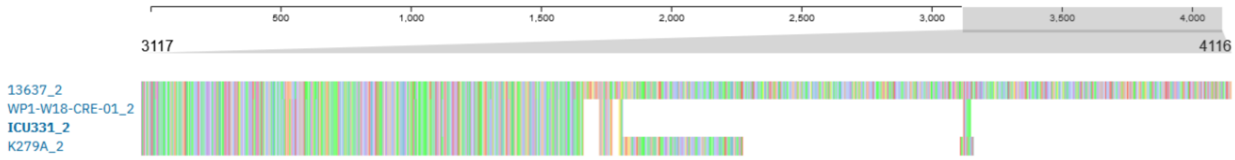

Supplementary Figure S1. MUSCLE alignment of the last ~1000 amino acids of the Type II-2 CdiA proteins from Smc strains 13637, WP1-W18-CRE-01, ICU331, and K279a. The full-length amino acid sequences of Type II-2 CdiA proteins from Smc strains 13637 (with putative rRNase activity), WP1-W18-CRE-01, ICU331, and K279a were aligned in MUSCLE and the alignment of the final ~1000 amino acids is shown.

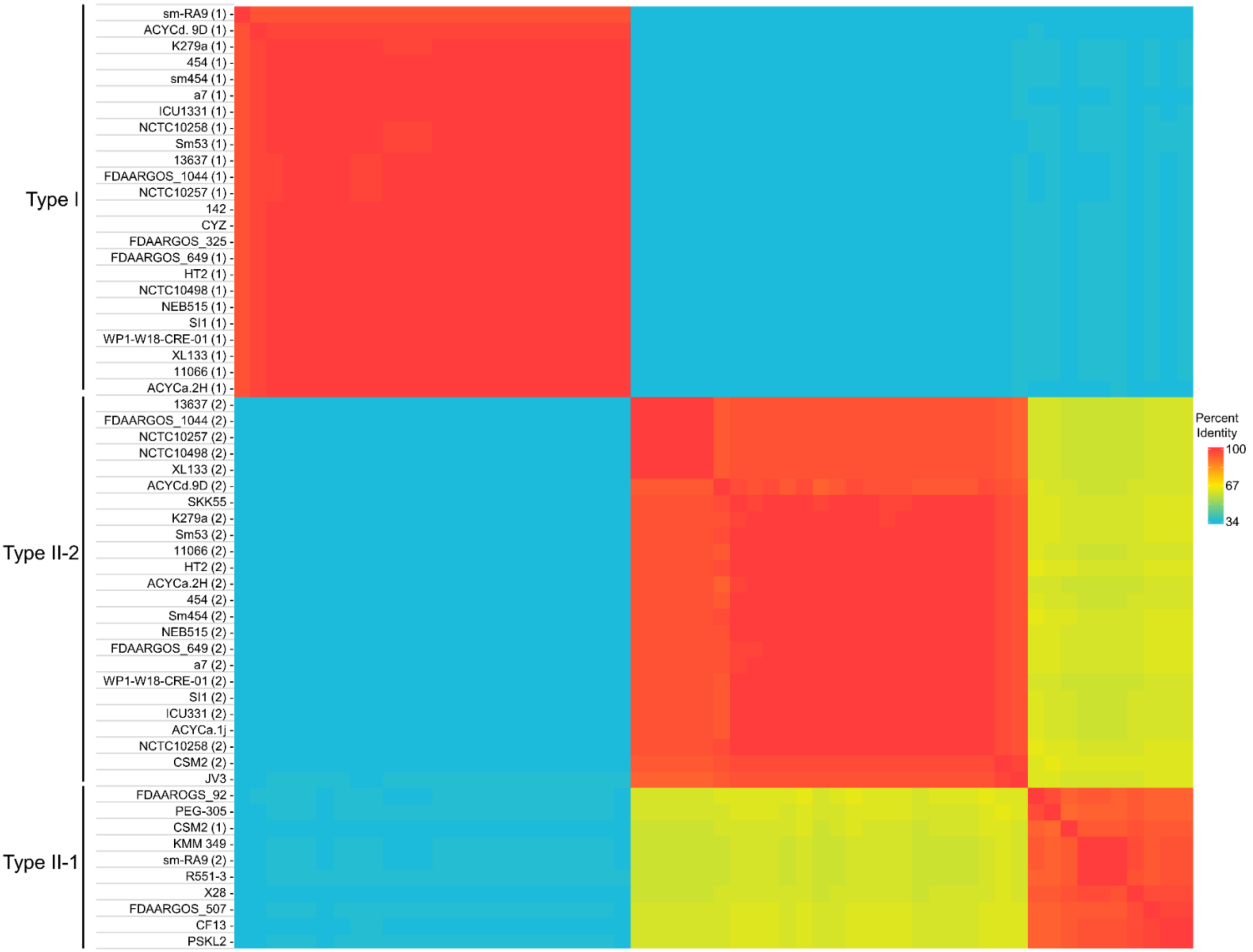

Supplementary Figure S2. Full length Smc CdiB proteins can be grouped into Type I, Type II-1, or Type II-2 based on their amino acid sequences. The amino acids of the full-length Smc CdiB proteins were obtained and aligned using Clustal Omega [1, 2]. A matrix was generated from the obtained percent identities in Heatmapper [3]. Strain names are depicted to the left. For strains that encode two CdiB proteins, numbers in parentheses next to the strain names indicate the specific CdiB number.

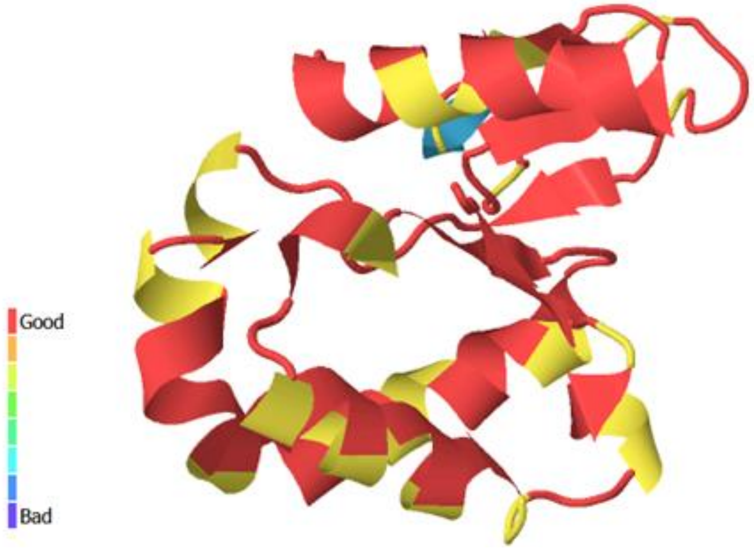

Supplementary Figure S3. Alignment of the predicted CdiA-CT^{Smc1} tertiary structure with CdiA-CT^{E479}. The tertiary structure of the final 150 amino acids of CdiA^{Smc1} was predicted using Phyre2 and the best hit (with CdiA-CT^{E479}) was visualized in Phyre2 Investigator [4]. The color scheme represents the alignment confidence between CdiA-CT^{Smc1} and CdiA-CT^{E479} [5, 6].

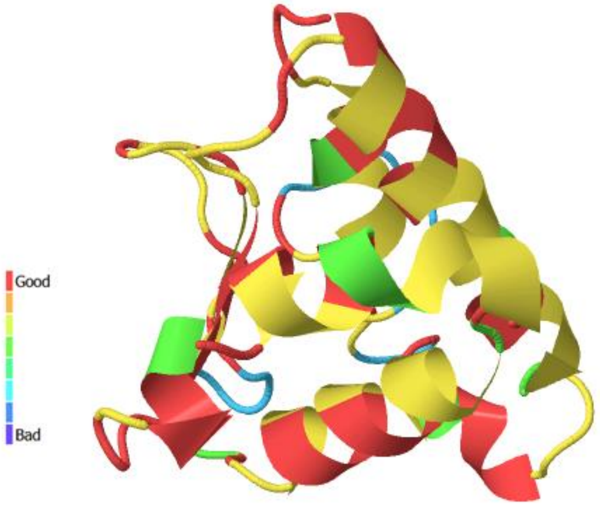

Supplementary Figure S4. Alignment of the predicted CdiI^{Smc1} tertiary structure with CdiI^{E479}. The tertiary structure of CdiI^{Smc1} was predicted using Phyre2 and the best hit (with CdiI^{E479}) was visualized in Phyre2 Investigator [4]. The color scheme represents the alignment confidence between CdiI^{Smc1} and CdiI^{E479} [5, 6].

References

1. **Sievers F, Higgins DG**. Clustal Omega for making accurate alignments of many protein sequences. *Protein Science* 2018;27:135–145.
2. **Madeira F, Madhusoodanan N, Lee J, Eusebi A, Niewielska A, *et al.*** The EMBL-EBI Job Dispatcher sequence analysis tools framework in 2024. *Nucleic Acids Res* 2024;gkae241.
3. **Babicki S, Arndt D, Marcu A, Liang Y, Grant JR, *et al.*** Heatmapper: web-enabled heat mapping for all. *Nucleic Acids Res* 2016;44:W147–W153.
4. **Kelley LA, Mezulis S, Yates CM, Wass MN, Sternberg MJE**. The Phyre2 web portal for protein modeling, prediction and analysis. *Nat Protoc* 2015;10:845–858.
5. **Johnson PM, Gucinski GC, Garza-Sánchez F, Wong T, Hung L-W, *et al.*** Functional Diversity of Cytotoxic tRNase/Immunity Protein Complexes from *Burkholderia pseudomallei* . *Journal of Biological Chemistry* 2016;291:19387–19400.
6. **Nikolakakis K, Amber S, Wilbur JS, Diner EJ, Aoki SK, *et al.*** The toxin/immunity network of *Burkholderia pseudomallei* contact-dependent growth inhibition (CDI) systems. *Mol Microbiol* 2012;84:516–529.
