# Supplementary material for: The dominant lineage of an emerging pathogen harbours contact-dependent inhibition systems
Source: Microb Genom. 2025 Jan 24;11(1):001332. doi: 10.1099/mgen.0.001332 (PMC11893273; doi:10.1099/mgen.0.001332)
Supplement: Uncited Table S1. [file mgen-11-01332-s002.pdf]

Supplementary Table S1. Details for the complete *Stenotrophomonas maltophilia* complex genomes used in this study.

Strain Name	Assembly Number	Genome Accession Number	Genome Coverage	Completeness (%)	Contamination (%)	Source	Year	Geographic Location
142	ASM2370249v1	GCA_023702495.1	100x	98.87	0.59	*Homo sapiens*	2022	China
454*	ASM2759480v1	GCA_027594805.1	143x	98.41	0.12	Cystic Fibrosis Patient	2023	Germany
1800	ASM91859399v1	GCA_918593995.1	136x	98.26	1.19	Industrial Effluent	2021	Algeria
11066	ASM3086704v1	GCA_030867045.1	100x	99.21	0.12	Sputum of Tuberculosis Patient	2023	China
13637**	ASM74299v1	GCA_000742995.1	417x	98.37	0.83	Oropharyngeal region of a patient with mouth cancer	2014	Unknown
a7	ASM3031599v1	GCF_030315995.1	100x	98.94	0.25	Amoeba	2023	China
AA1	ASM202560v1	GCF_002025605.1	136.8x	95.55	4.94	*Zea mays* root	2017	USA
AB550	ASM218954v2	GCA_002189545.2	306x	97.94	1.32	Water	2018	Australia
ACYCa.1J	ASM2064107v2	GCA_020641075.2	75x	99.12	0.6	Freshwater Stream	2022	Hong Kong
ACYCa.2H	ASM2064145v2	GCA_020641455.2	35x	99	0.73	Freshwater Stream	2022	Hong Kong
ACYCb.1K	ASM2064141v2	GCA_020641415.2	91x	99.47	0.63	Freshwater Stream	2022	Hong Kong
ACYCb.6H	ASM2064142v2	GCA_020641425.2	342x	99.29	0.82	Freshwater Stream	2022	Hong Kong
ACYCb.10K	ASM2064136v2	GCA_020641365.2	75x	99.22	1.02	Freshwater Stream	2022	Hong Kong
ACYCc.3B	ASM2064109v2	GCA_020641095.2	79x	98.11	0.16	Freshwater Stream	2022	Hong Kong
ACYCd.9D	ASM2064108v2	GCA_020641085.2	340x	99.16	0.6	Freshwater Stream	2022	Hong Kong
CF13	ASM1297176v1	GCA_012971765.1	30x	99	0.33	Sputum of Cystic Fibrosis Patient	2020	Australia
Col1	ASM1904890v1	GCF_019048905.1	145x	98.43	0.52	Pavement Soil	2021	South Korea
CPBW01	ASM985717v1	GCA_009857175.1	634x	98.6	0.93	*Leptinotarsa decemlineata* (Colorado potato beetle)	2020	China
CSM2	ASM284738v1	GCA_002847385.1	150x	98.48	0.56	Laboratory Sink	2017	Mexico
CYZ	ASM2642829v1	GCF_026428295.1	174.7x	98.76	0.17	Patient Sputum	2022	China
D457	ASM28459v1	GCA_000284595.1	67x	98.38	0.35	Patient	2012	Spain
DHHJ	ASM1665918v1	GCA_016659185.1	336x	98.47	0.49	Soil	2021	China
FDAARGOS_92	ASM295111v1	GCA_002951115.1	20.8x	98.61	0.45	Patient Respiratory Culture	2018	USA
FDAARGOS_325	ASM220888v2	GCA_002208885.2	15x	98.91	0.78	Patient Eye	2018	USA
FDAARGOS_507	ASM381298v1	GCA_003812985.1	19.2x	98.83	0.63	Sputum of Cystic Fibrosis Patient	2018	Unknown
FDAARGOS_649	ASM869398v1	GCA_008693985.1	676.4x	98.51	0.3	*Homo sapiens*	2019	USA
FDAARGOS_1044**	ASM1612693v1	GCA_016126935.1	717.6x	98.37	0.83	Unknown	2020	USA
FZD2	ASM2327752v1	GCA_023277525.1	340x	97.86	2.07	Activated Sludge from Sewage Treatment Plant	2022	Poland
HT2	ASM2889144v1	GCA_028891445.1	333x	98.96	0.69	Rhizosphere of *Bidens alba*	2023	Hong Kong
HW002Y	ASM2520082v1	GCA_025200825.1	231x	97.43	0.3	Tap Water from Hospital Intensive Care Unit Ward	2022	Malaysia
ICU331	ASM967660v1	GCA_009676605.1	150x	98.77	0.85	Unknown	2019	Germany
ISMMS2***	ASM127465v1	GCA_001274655.1	160x	98.43	0.27	Patient Blood	2015	USA
ISMMS2R***	ASM127467v1	GCA_001274675.1	403x	98.43	0.27	Patient Blood	2015	USA
ISMMS3	ASM127459v1	GCA_001274595.1	153x	97.66	1.89	Patient Blood	2015	USA
JV3	ASM22388v1	GCA_000223885.1	30x	98.23	0.72	Plants	2011	Brazil
JZL8	ASM2013208v1	GCF_020132085.1	240x	98.47	0.27	Clivia Plant	2021	China
K279a	ASM7248v1	GCA_000072485.1	11.1x	99.25	0.17	Blood From Cancer Patient	2008	United Kingdom
KMM 349	ASM961803v1	GCA_009618035.1	351x	98.94	0.25	Marine Sponge	2019	Philippine Sea
LH-B2	ASM2945743v1	GCF_029457435.1	92x	94.44	0.49	Petroleum Contaminated Soil	2023	China
MER1	ASM1106722v1	GCA_011067225.1	463x	97.32	1.06	Wastewater	2020	China
NCTC10257**	49243_F02	GCA_900186865.1	156x	98.37	0.83	Oropharyngeal region of a patient with mouth cancer	2017	Unknown
NCTC10258	44087_C01	GCA_900475405.1	100x	99.12	0.15	Patient Cerebrospinal Fluid	2018	Unknown
NCTC10259	44982_B01	GCA_900636905.1	100x	99.02	0.74	Unknown	2018	Unknown
NCTC10498	46463_B01	GCA_900475685.1	100x	99.46	0.39	Human Oropharyngeal Swab	2018	Unknown
NCTC13014	43781_D01	GCA_900636655.1	100x	98.41	1.39	Unknown	2018	Unknown
NEB515	ASM1264702v1	GCA_012647025.1	75.6x	98.67	0.26	Water	2020	Unknown
OUC_Est10	ASM213841v1	GCA_002138415.1	123x	98.86	0.87	Soil	2017	China

PEG-42	ASM967652v1	GCA_009676525.1	150x	98.24	0.71	Human Urine	2019	Switzerland
PEG-68	ASM967648v1	GCA_009676485.1	150x	99.16	1.12	Patient Sputum	2019	Austria
PEG-141	ASM967658v1	GCA_009676585.1	150x	95.44	1.38	Patient Respiratory Tract	2019	Germany
PEG-173	ASM967656v1	GCA_009676565.1	150x	98.34	1.74	Patient Respiratory Tract	2019	Germany
PEG-305	ASM967654v1	GCA_009676545.1	150x	98.67	1.22	Patient Wound	2019	Germany
PEG-390	ASM967650v1	GCA_009676505.1	150x	98.88	0.77	Patient Respiratory Tract	2019	Germany
PSKL2	ASM2201473v1	GCA_022014735.1	76x	98.62	0.93	Storm Drain Water	2022	Hong Kong
R551-3	ASM2066v1	GCA_000020665.1	17.6x	99.02	0.12	Poplar Plant	2008	Germany
SG.Y2	ASM2564225v1	GCA_025642255.1	85x	95.4	0.93	*Sempervivum tectorum* Plant	2022	Hong Kong
SI1	ASM3240120v1	GCA_032401205.1	567x	98.99	1.05	Heavy Metal Contaminated Soil	2023	Pakistan
SJTH1	ASM300643v1	GCA_003006435.1	188x	98.34	1.06	Wastewater	2018	China
SJTL3	ASM320583v1	GCA_003205835.1	249x	98.21	2.37	Wastewater	2018	China
SKK55	ASM967646v1	GCA_009676465.1	150x	98.48	1.69	Patient Respiratory Sample	2019	Germany
SM 866	ASM783365v1	GCA_007833655.1	60x	96.73	1.72	Intensive Care Patient Blood	2019	India
Sm53	ASM967640v1	GCA_009676405.1	150x	99.16	0.69	Patient Sputum	2019	Germany
sm454*	ASM967642v1	GCA_009676425.1	150x	98.86	0.38	Patient Sputum	2019	Germany
sm-RA9	ASM967644v1	GCA_009676445.1	150x	98.39	0.88	Sewage	2019	Germany
STEN00241	ASM3309576v1	GCF_033095765.1	84x	98.3	1.33	Human Sputum	2023	USA
T50-20	ASM1407653v1	GCF_014076535.1	100x	98.01	0.81	Biofilm Reactor	2020	China
U5	ASM967638v1	GCA_009676385.1	150x	97.23	1.3	Environmental	2019	Germany
W18	ASM303098v1	GCA_003030985.1	40.9x	98.21	0.76	Soil	2018	China
WGB211	ASM2144186v1	GCF_021441865.1	344x	97.69	1.03	Shale	2022	China
WP1-W18-CRE-01	ASM1415697v1	GCA_014156975.1	398x	98.96	1.14	Wastewater	2020	Japan
X28	ASM697412v1	GCF_006974125.1	56.5x	98.53	0.61	Aerobic Granular Sludge	2019	China
XL133	ASM2008008v1	GCA_020080085.1	100x	99.18	0.63	Cucumber Rhizosphere	2021	China
ZT1	ASM2137833v1	GCA_021378335.1	700x	99.05	0.73	Bile Acid of Cholelithiasis Patient	2022	China

* These genomes likely belong to the same *Stenotrophomonas maltophilia* complex strain but have unique accession numbers and were sequenced by different entities.

** These genomes likely belong to the same *Stenotrophomonas maltophilia* complex strain but have unique accession numbers and were sequenced by different entities.

*** These genomes belong to two *Stenotrophomonas maltophilia* complex isolates from the same patient isolated within ten days.

Supplementary Table S2. Smc CdiA protein amino acid lengths.

Name	Amino Acids	Type
sm-RA9 (1)	3427	I
FDAARGOS_649 (1)	3436	I
ACYCa.2H (1)	3436	I
WP1-W18-CRE-01 (1)	3436	I
NEB515 (1)	3467	I
ACYCd.9D (1)	3414	I
NCTC10258 (1)	3429	I
11066 (1)	3429	I
454 (1)	3429	I
sm454 (1)	3429	I
FDAARGOS_325	3429	I
HT2 (1)	3512	I

NCTC10498 (1)	3512	I
XL133 (1)	3512	I
142	3512	I
a7 (1)	3512	I
CYZ	3512	I
SI1 (1)	3512	I
ICU331 (1)	3497	I
Sm53 (1)	3497	I
13637 (1)	3497	I
FDAARGOS_1044 (1)	3497	I
NCTC10257 (1)	3497	I
K279a (1)	3497	I
PEG-305	4574	II-1
R551-3	4966	II-1
KMM349	4966	II-1
Sm-RA9 (2)	4966	II-1
CF13	5036	II-1
X28	5036	II-1
FDAARGOS_92	5174	II-1
CSM2 (1)	5157	II-1
FDAARGOS_507	5155	II-1
PSKL2	5160	II-1
454 (2)	4109	II-2
Sm454 (2)	4109	II-2
CSM2 (2)	4223	II-2
ACYCd.9D (2)	4093	II-2
ICU331 (2)	3542	II-2
K279a (2)	3658	II-2
SKK55	4191	II-2
ACYCa.1j	4112	II-2
NCTC10498 (2)	4097	II-2
13637 (2)	4097	II-2
FDAARGOS_1044 (2)	4097	II-2
NCTC10257 (2)	4097	II-2
XL133 (2)	4097	II-2
Sm53 (2)	4108	II-2
NEB515 (2)	4229	II-2
NCTC10258 (2)	4269	II-2
ACYCa.2H (2)	4114	II-2
FDAAROGS_649 (2)	4130	II-2
WP1-W18-CRE-01 (2)	3537	II-2
JV3	3729	II-2
a7 (2)	3756	II-2
SI1 (2)	3723	II-2
11066 (2)	3723	II-2
HT2 (2)	3723	II-2

Supplementary Table S3. Details for plasmids used in this study.

Plasmid Name	Description	Source
pET-28a(+)	Bacterial expression vector with T7 promoter	Novagen
pCCV174	pET-28a(+) with *cdiA-CT* (last 900 nucleotides of the *cdiA* gene) from CCV131 downstream of the T7 promoter	This study
pCCV192	pET-28a(+) with *cdiA-CT* (last 900 nucleotides of the *cdiA* gene) and the *cdiI* gene from CCV131 downstream of the T7 promoter	This study
pCCV205	pCCV174 with the E3384A mutation in *cdiA*-CT	This study
pCCV206	pCCV174 with the D3412A mutation in *cdiA*-CT	This study
pCCV207	pCCV174 with the D3426A mutation in *cdiA*-CT	This study
pCCV208	pCCV174 with the H3456A mutation in *cdiA*-CT	This study

Supplementary Table S4. Details for primers used in this study.

Primer Name	Primer Sequence	Description
oCCV059	TAAGAAGGAGATATACCATGCGCT TCGAGTTCCAGGAG	Forward primer to amplify *cdiA-CT*^{Smc1} from CCV131 and insert into pET-28a(+) to create pCCV174.
oCCV060	CAGCTTCCTTTCGGGCTTTGTTAC CTCAAAATAATAATTTTACCCTGC	Reverse primer to amplify *cdiA-CT*^{Smc1} from CCV131 and insert into pET-28a(+) to create pCCV174.
oCCV061	AAATTATTATTTTGAGGTAACAAAG CCCGAAAGGAAGCTGAG	Forward primer to amplify the pET-28a(+) construct and insert *cdiA-CT*^{Smc1} from CCV131 to create pCCV174.
oCCV062	GGCTCCTGGAACTCGAAGCGCAT GGTATATCTCCTTCTTAAAGTTAAA CAAAATTATTTCTAGAGG	Reverse primer to amplify the pET-28a(+) construct and insert *cdiA-CT*^{Smc1} from CCV131 to create pCCV174.
oCCV089	GCCCGAAAGGAAGCTGAGTTAAG GAGATATACCATGGCGATGTACAT TTCTATTGGC	Forward primer to amplify *cdiI-CT*^{Smc1} from CCV131 and insert into pCCV174 to create pCCV192.
oCCV090	GCTCAGCGGTGGCAGCAGCCTTA CGCGGACTCCCGATAAC	Reverse primer to amplify *cdiI-CT*^{Smc1} from CCV131 and insert into pCCV174 to create pCCV192.
oCCV091	GTTATCGGGAGTCCGCGTAAGGC TGCTGCCACCG	Forward primer to amplify the pCCV174 backbone and insert *cdiI-CT*^{Smc1} from CCV131 to create pCCV192.
oCCV092	ATAGAAATGTACATCGCCATGGTA TATCTCCTTAACTCAGCTTCCTTT CGGG	Reverse primer to amplify the pCCV174 backbone and insert *cdiI-CT*^{Smc1} from CCV131 to create pCCV192.
oCCV095	CATGCGGGTCAGGCGGCCGT	Forward primer to introduce the E3384A mutation in the *cdiA-CT*^{Smc1} sequence from plasmid pCCV174.
oCCV096	CACTGCAATTTTCCCCTTATCCG	Reverse primer to introduce the E3384A mutation in the *cdiA-CT*^{Smc1} sequence from plasmid pCCV174.
oCCV097	TCCCGCGTTCGTATTTGTCAGTG GGCC	Forward primer to introduce the D3412A mutation in the *cdiA-CT*^{Smc1} sequence from plasmid pCCV174.
oCCV098	TTTTTACTGCCCTGCACCG	Reverse primer to introduce the D3412A mutation in the *cdiA-CT*^{Smc1} sequence from plasmid pCCV174.
oCCV099	GTGGCGTTCATGTGGACAGACGG TACAA	Forward primer to introduce the D3426A mutation in the *cdiA-CT*^{Smc1} sequence from plasmid pCCV174.

oCCV100	GGTCTTCCCCGGATAGGG	Reverse primer to introduce the D3426A mutation in the *cdiA-CT*^{Smc1} sequence from plasmid pCCV174.
oCCV101	GATGCGGTAAACAAGGCGGATATGGTTCC	Forward primer to introduce the H3456A mutation in the *cdiA-CT*^{Smc1} sequence from plasmid pCCV174.
oCCV102	TATCAGCTGCCGCTGATTCT	Reverse primer to introduce the H3456A mutation in the *cdiA-CT*^{Smc1} sequence from plasmid pCCV174.
